# Herpes simplex virus type 1 UL14 tegument protein regulates intracellular compartmentalization of major tegument protein VP16

**DOI:** 10.1186/1743-422X-8-365

**Published:** 2011-07-26

**Authors:** Akane Ohta, Yohei Yamauchi, Yoshifumi Muto, Hiroshi Kimura, Yukihiro Nishiyama

**Affiliations:** 1 Department of Virology, Graduate School of Medicine, Nagoya University, Tsurumai-cho 65, Showa-ku, Nagoya 466-8550, Japan; 2 ETH Zurich, Institute of Biochemistry, Schafmattstrasse 18, HPM, ETH Hönggerberg, Zurich, Switzerland

**Keywords:** Herpes simplex virus, UL14, VP16, molecular chaperone, heat shock proteins, aggresome

## Abstract

**Background:**

Herpes simplex virus type 1 (HSV-1) has a complicated life-cycle, and its genome encodes many components that can modify the cellular environment to facilitate efficient viral replication. The protein UL14 is likely involved in viral maturation and egress (Cunningham C. et al), and it facilitates the nuclear translocation of viral capsids and the tegument protein VP16 during the immediate-early phase of infection (Yamauchi Y. et al, 2008). UL14 of herpes simplex virus type 2 exhibits multiple functions (Yamauchi Y. et al, 2001, 2002, 2003).

**Methods:**

To better understand the function(s) of UL14, we generated VP16-GFP-incorporated UL14-mutant viruses with either single (K51M) or triple (R60A, R64A, E68D) amino acid substitutions in the heat shock protein (HSP)-like sequence of UL14. We observed the morphology of cells infected with UL14-null virus and amino acid-substituted UL14-mutant viruses at different time points after infection.

**Results:**

UL14(3P)-VP16GFP and UL14D-VP16GFP (UL14-null) viruses caused similar defects with respect to growth kinetics, compartmentalization of tegument proteins, and cellular morphology in the late phase. Both the UL14D-VP16GFP and UL14(3P)-VP16GFP viruses led to the formation of an aggresome that incorporated some tegument proteins but did not include nuclear-egressed viral capsids.

**Conclusions:**

Our findings suggest that a cluster of charged residues within the HSP-like sequence of UL14 is important for the molecular chaperone-like functions of UL14, and this activity is required for the acquisition of functionality of VP16 and UL46. In addition, UL14 likely contributes to maintaining cellular homeostasis following infection, including cytoskeletal organization. However, direct interactions between UL14 and VP16, UL46, or other cellular or viral proteins remain unclear.

## Background

Herpes simplex virus (HSV) type 1 (HSV-1) is a large, enveloped DNA virus whose genome contains at least 74 genes [1,2]. Previous studies showed that approximately half of these genes are unessential for virus replication in cell culture [2]. However, the dispensable gene products may be important for viral growth and propagation *in vivo *[3]. Additionally, 40 core genes have been identified based on significant genomic homology among the three Herpesviridae subfamilies[4]. However, one-third of the core genes are dispensable[3], and their functions remain unknown. To gain a comprehensive understanding of the HSV lifecycle, it is important to investigate the functions of these accessory genes both *in vitro *and *in vivo*.

**Figure 1:**
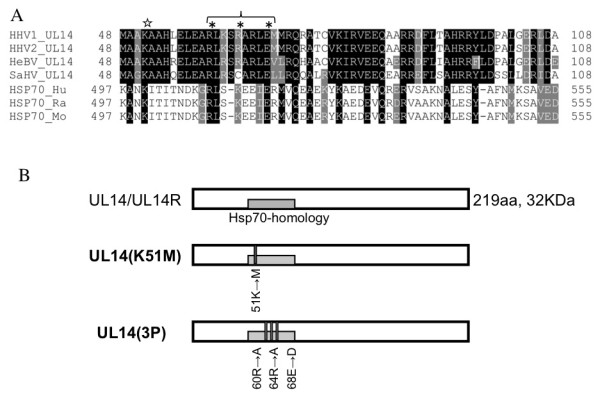
**Generation of UL14 mutant viruses**. (A) Comparison of the amino acid (aa) sequences of UL14 (residues 48-108) of simplex type herpesviruses (human herpesvirus type 1 (HHV1), human herpesvirus type 2 (HHV2), herpesvirus B virus (HeBV), and Saimiriine herpesvirus 1 (SaHV)) and Hsp70 (residues 497-555) of human Hsp70 (HSP70_Hu), Rat Hsp70 (HSP70_Ra), and mouse Hsp70 (HSP70_Mo). The letters shown in black are identical to those of HHV-1 UL14, and the letters shown in gray are similar residues. The star indicates residue K51. The asterisks indicate three residues that are substituted in UL14(3P). (B) A schematic diagram of the UL14 wild-type (219 aa) and amino acid-substituted mutant proteins. The gray box indicates the HSP-like homology domain of UL14 (aa 48-108), and the thin black bars show the substituted residues introduced in this study.

UL14 is an established core gene but it is dispensable for viral replication *in vitro*. UL14 is a 32-kDa protein expressed in the late phase of viral infection, after viral DNA synthesis has occurred [5,6]. The coding region of HSV-1 UL14 overlaps that of UL13, but most conserved residues are located in the nonoverlapping region. The overlapping region encodes a variable-length C-terminal domain that is poorly conserved [6]. We previously showed that the UL14 protein of HSV type 2 (HSV-2) facilitates the translocation of both capsid protein VP26 (which is encoded by the UL35 gene) and UL33 protein into the nucleus in cells co-expressing these proteins in the absence of viral infection[7]. UL14 also exhibits heat shock protein (HSP)-like functions when expressed alone in cells. Additionally, a region that is highly conserved between HSV-1 and HSV-2, especially _60_RLKSRARLE_68_, is similar to an α-helix contained within the substrate-binding region of Hsp70 (Figure 1A) [8,9]. UL14 undergoes intercellular trafficking and prevents apoptosis induced by chemical and hypertonic stress [10,11]. Finally, upon infection, UL14 is incorporated into the virion as a minor tegument protein, and it is required for efficient growth of the virus [5,6].

VP16 is an abundant 65-kDa phosphoprotein that is synthesized during the late phase of infection, and it is subsequently packaged into virions as a major tegument protein [12-15]. VP16 acts during the earliest stages of infection in concert with HCF-1 and Oct-1 to induce transcription of the viral immediate-early (IE) genes, thereby facilitating the onset of lytic gene expression (reviewed in references [2,16]). Incorporation of UL14 into the virion tegument promotes the efficient transport of virion-associated VP16 into the nucleus immediately after virus infection [2], and deletion of UL14 significantly alters the targeting of viral capsids to the nuclear pore. The combination of these factors likely contributes to the observed 4-fold delay in the initial expression of the IE genes encoded by ICP0 and ICP4 when cells are infected with UL14-deleted (UL14D) virus [17].

In the present study, we wished to determine whether the HSP-like sequence of UL14 mediated the nuclear transport of VP16 during the early phase of infection. However, we unexpectedly found that these residues were important for viral replication in the late phase of infection, rather than in the IE phase of infection.

## Results

### Construction of UL14 mutant viruses with amino acid substitutions

To analyze the functions of the UL14 tegument protein, we constructed two mutant viruses with either single or triple amino acid substitutions in UL14. We previously identified a region of homology between the C-terminal domain of mammalian Hsp70 and the N-terminal region of HSV UL14 [9]. The C-terminal region of Hsp70 is thought to modulate substrate recognition and/or maintain its substrate-binding state [18]. Figure 1A shows the sequence alignment between mammalian Hsp70 and herpes virus UL14. K51 is conserved in both α-herpes viruses and the sequence _60_RLKSRARLE_68 _of UL14, which contains a cluster of charged residues, and this region is highly similar to Hsp70 [6,9]. We also incorporated green fluorescence protein (GFP)-conjugated VP16 (VP16GFP) into the genome of these newly generated UL14-mutant viruses (Figure 1B). Successful incorporation of the mutations was verified by sequence analysis. The viruses used in this study were as follows: 1) UL14D-VP16GFP (UL14-null mutant); 2) UL14R-VP16GFP (UL14D-rescued virus); 3) UL14(K51M)-VP16GFP (K51 to methionine substitution); 4) UL14(3P)-VP16GFP (R60, R64 to alanine and E68 to aspartic acid substitutions); and 5) UL14(3P)R-VP16GFP (UL14(3P)-rescued virus).

### Growth properties of UL14 mutant viruses

**Figure 2:**
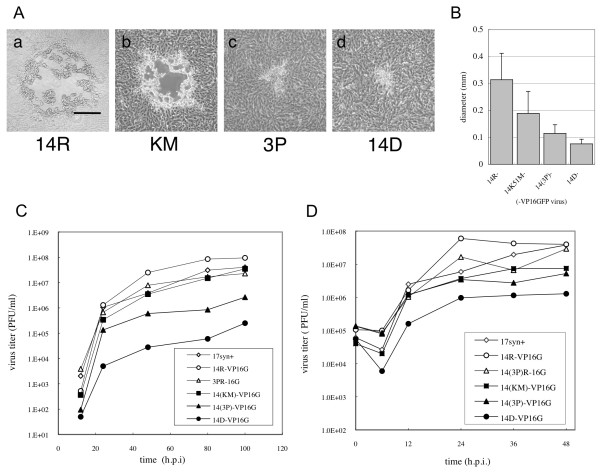
**Growth properties of new UL14 mutant viruses**. (A) Plaque morphology of the viruses used in the study. Vero cells were infected with (a) UL14R-VP16GFP, (b) UL14K51M-VP16GFP, (c) UL14(3P)-VP16GFP, (d) UL14D-VP16GFP at a low MOI and maintained in medium containing γ-globulin for 72 hours. The bar in panel B represents 0.5 mm. (B) Quantification of plaque size. Cells were infected at a low MOI with the indicated viruses in the presence of γ-globulin and examined at 36 h.p.i. Thirty plaques or more were randomly selected and their diameters were measured under a Nikon microscope equipped with a CCD camera. The bar and whisker for each show the average diameter of the plaques and the standard deviation, respectively. There was a significant difference between the sizes of the plaques formed by 14R- and UL14(3P)-VP16GFP viruses (p < 0.001; *t*-test). (C) After the cells were infected with viruses at an MOI of 0.001, multi-step growth curves were examined. At 12, 24, 48, 80, 100 h.p.i. cells and media were harvested together, frozen, and subsequently thawed for titration on Vero cell monolayers. (D) The one-step growth curves of the strains examined after the viruses were infected at an MOI of 3.0. At 0, 6, 12, 24, 36, 48 h.p.i. cells and media were harvested together, frozen, and thawed for titration on Vero cell monolayers.

UL14-null virus and mutant viruses all exhibited delayed viral growth in Vero cells. However, the sizes of the plaques differed considerably. We compared the diameters of the plaques formed around the different groups of Vero cells at 72 hours post-infection (h.p.i.) (Figure 2A). UL14R- and UL14(K51M)-VP16GFP formed similar medium-sized plaques, whereas those formed by UL14(3P)- and UL14D-VP16GFP were smaller. We quantified the diameter of at least 30 plaques for each virus at 36 h.p.i., and there was a significant difference between the plaque sizes of UL14R- and UL14(3P)-VP16GFP viruses (p < 0.001; *t*-test) (Figure 2B).

To analyze the impact of these point mutations on viral growth, we investigated both multiple-step and single-step growth kinetics. At a multiplicity of infection (MOI) of 0.001 (PFU/cell), UL14(K51M)-VP16GFP exhibited a 50% decrease in growth compared to that of the wild-type virus at a viral yield of 100 h.p.i. Moreover, UL14(3P)- and UL14D-VP16GFP viruses revealed further declines of 1-log and 2-logs, respectively (Figure 2C). At an MOI of 3, UL14(K51M)- and UL14(3P)-VP16GFP exhibited decreased infection of almost 1-log compared to the wild-type viruses, but these decreases were not as large as that exhibited by UL14D-VP16GFP (Figure 2D). These results suggest that both the K51M and the triple mutants affected single- and multiple-growth kinetics, although the three-point mutation had a more severe impact on viral growth compared to the K51M mutation alone.

### Mutation of UL14 altered the intracellular compartmentalization of newly synthesized VP16GFP

The delayed viral growth exhibited by UL14D- and UL14(3P)-VP16GFP suggest that mutation of UL14 reduces the efficiency of viral replication and/or propagation in infected cells. We next investigated the intracellular localization of the viral components that comprise viral particles. VP16 is a major tegument protein abundantly expressed in infected cells, and we examined the localization of VP16GFP in HEp-2 cells infected with the different UL14 mutant viruses at an MOI of 3.

**Figure 3:**
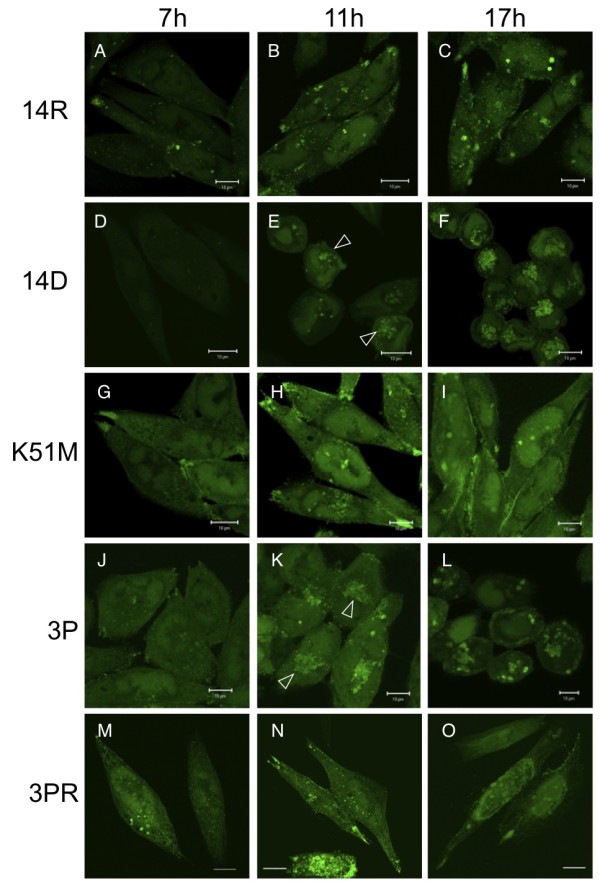
**Intracellular localization of VP16GFP in cells infected with UL14-mutant viruses**. HEp-2 cells were infected with the virus at a MOI of 3 PFU/cell with the following viruses: (A-C) UL14R-VP16GFP, (D-F) UL14D-VP16GFP, (G-I) UL14(K51M)-VP16GFP, (J-L) UL14(3P)-VP16GFP, (M-O) UL14(3P)R-VP16GFP. The cells were then fixed at 7, 11, and 17 h.p.i. and examined using laser confocal microscopy (Zeiss LSM510). UL14D-VP16GFP infection induced prominent cell rounding and the accumulation of VP16GFP at the periphery of the nucleus (E, F). This phenotype, although less distinct, was observed in cells infected with UL14(3P)-VP16GFP, especially at later time points after infection (K, L). Open arrows indicate clumps of VP16GFP that were readily observed (H, K). All images were acquired with the same magnification. Scale bars: 10 μM.

Wild-type virus infection led to both cytoplasmic and nuclear localization of VP16GFP (Figure 3A-C). VP16GFP showed a diffuse nuclear pattern followed by the formation of discrete subnuclear foci that grew larger at later times after infection. In the cytoplasm, we observed both diffuse and dotted patterns. Overall, the distribution of VP16 in cells infected with wild-type virus was comparable to that previously reported [19]. Cells infected with UL14(K51M)-VP16GFP or UL14(3P)R-VP16GFP showed a phenotype similar to wild-type virus; however, subnuclear foci were not clearly visualized. There were no substantial differences in the localization of VP16GFP in the mutant virus infected cells compared to wild-type cells (Figure 3G-I, MO).

Cells infected with UL14D-VP16GFP showed a distinct overall morphology and distribution of VP16GFP (Figure 3D-F). At 7 h.p.i, VP16GFP showed diffuse nuclear and cytoplasmic distribution similar to that seen in wild-type-infected cells (Figure 3D). At later time points, VP16GFP had accumulated in the perinuclear region in clumps forming a globular structure (Figure 3E). At 17 h.p.i. VP16GFP was found in the nucleus and the perinuclear region in larger aggregates, and the triple mutant exhibited a phenotype similar to that of UL14D-VP16GFP (Figure 3J-L). Additionally, VP16GFP began to accumulate at the nuclear periphery at later times post-infection.

### Intracellular localization of other viral proteins in UL14-mutant viruses

**Figure 4:**
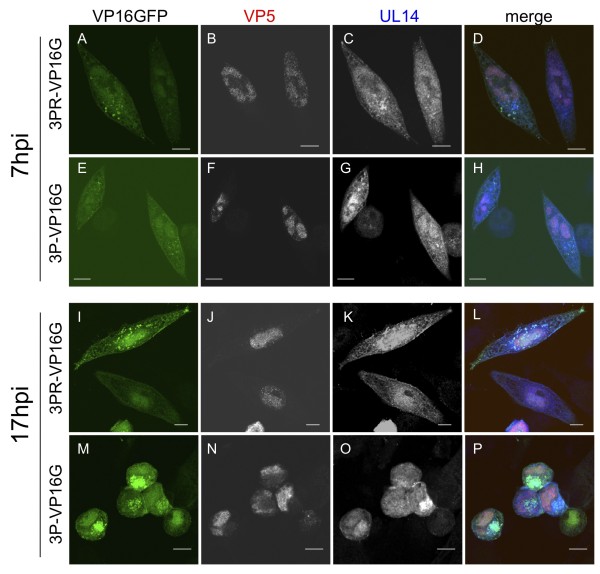
**Localization of triple mutant UL14 proteins and VP5 in infected cells**. The localization of wild-type and mutant UL14 proteins and capsid VP5 protein was observed in HEp-2 cells infected with UL14(3P)-VP16GFP or UL14(3P)R-VP16GFP viruses. Cells were infected at an MOI of 3, fixed at 11 h.p.i., permeabilized, and stained with either an anti-HSV-1 UL14 rabbit polyclonal antiserum (diluted 1:1000) [6], or an anti-HSV1+HSV2 ICP5 mouse monoclonal antibody (diluted 1:200).

We next investigated the localization of other viral proteins in cells infected with UL14D-, UL14R-, or UL14(3P)-VP16GFP. Immunofluorescence analysis revealed that the capsid protein VP5 predominantly localized to the nucleus from 7 to 17 h.p.i in cells infected with wild-type virus (Figure 4). Moreover, there was little difference in the compartmentalization of VP5 between cells infected with UL14(3P)R- or UL14(3P)-VP16GFP viruses. Both wild-type UL14 and triply mutated UL14(3P) localized to the nucleus and cytoplasm at 7 h.p.i, and the largest population was seen in the nucleus. Wild-type UL14 in UL14(3P)R-infected cells was distributed throughout the cell at 17 h.p.i., but it was predominantly localized to the nucleus. However, the distinct intracellular localization pattern exhibited by UL14(3P) was also seen in UL14(3P)-16GFP-infected cells at 17 h.p.i. (Figure 4). In both cases, there was the accumulation and aggregation of VP16GFP at the perinuclear region.

**Figure 5:**
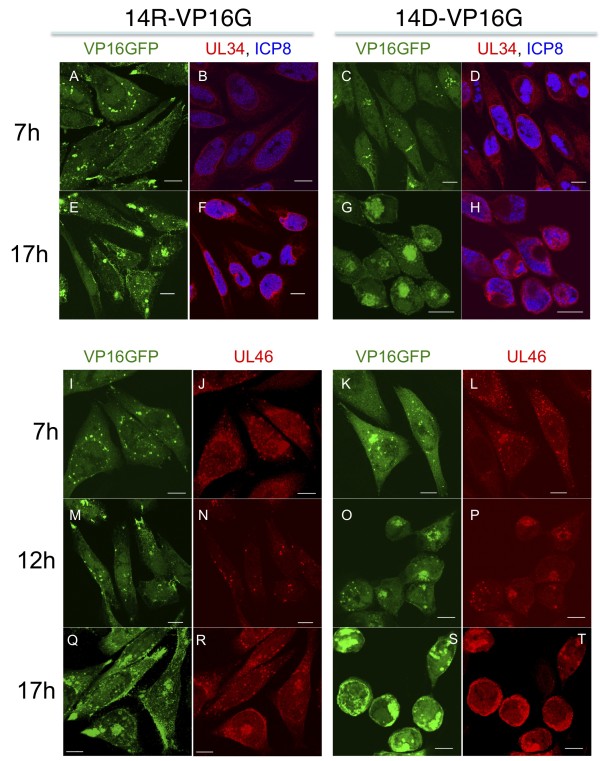
**Intracellular localization of viral non-structural or tegument proteins**. (A-H) Comparison of localization among VP16GFP, ICP8, and UL34 in UL14R- or UL14D-VP16GFP-infected cells. HEp-2 cells were infected at an MOI of 3, fixed at 7 and 17 h.p.i., permeabilized, and treated with anti-ICP 8 and anti-UL34 rabbit polyclonal antisera (diluted 1:1000) [21]. The structures of the replication compartments represented by ICP8 did not differ between cells infected with UL14R-VP16GFP (B, F) and UL14D-VP16GFPF (D, H) at the two time points. The inner nuclear membrane represented by UL34 staining revealed an apparent dent in the nucleus only in UL14D-infected cells, and this was accompanied by the formation of the VP16GFP aggregates (H).

We subsequently used immunofluorescence analysis to evaluate the localization of two non-structural viral proteins, ICP8 and UL34. ICP8 is a single-stranded DNA-binding protein, and localizes to sites of viral replication in the cell nucleus [20]. UL34 protein is a tail-anchored type II membrane protein that localizes to the inner nuclear membrane, and it is required for the efficient envelopment of progeny virions at the nuclear envelope [21,22]. ICP8 localized to small punctate foci in the nucleus at 7 h.p.i. in UL14R-VP16GFP-infected cells, and, by 17 h.p.i. ICP8 localized to a large globular structure (Figure 5). In cells infected with UL14D-VP16GFP, the localization of ICP8 was similar to that seen in UL14R-VP16GFP-infected cells at both time points. In cells infected with either UL14R- or UL14D-VP16GFP, UL34 localized to the inner nuclear membrane (Figure 5), and we confirmed the localization of UL34 by co-staining with anti-lamin A/C (data not shown). Additionally, there was a dramatic morphological change in the nuclei of UL14D-VP16GFP-infected cells, which was accompanied by the formation of the VP16GFP aggregates. These observations suggest that UL14 does not play an important role in the formation of replication compartments in the nucleus. However, the loss of UL14 or expression of UL14 mutants affected the cytoplasmic environment during viral replication.

### A UL46 tegument protein (VP11/12) localized to the sites of VP16GFP accumulation

We also investigated the localization of UL46, which strongly associates with VP16 (UL48 protein) and UL47 in the HSV virion [23]. In cells infected with UL14R-VP16GFP, a substantial fraction of UL46 localized to cytoplasmic face of the nucleus at 7, 12, and 17 h.p.i., as previously reported (Figure 5). The cytoplasmic fraction of UL46 of UL14R-VP16GFP colocalized with VP16GFP around the cell nucleus. Although VP16GFP was also localized to the periphery of the cell, UL46 associated with UL14R-VP16GFP did not localize to that area. In cells infected with UL14D-VP16GFP, the localization of UL46 at 7 h.p.i. was similar to that seen in the UL14R-infected cells. However, at 12 h.p.i. the dotted distribution pattern of UL46 in UL14D-VP16GFP-infected cells seen early after infection changed, and UL46 became aggregated at the perinuclear or cytoplasmic space.

### Cellular morphological alterations were detected in the UL14-defective virus-infected cells

**Figure 6:**
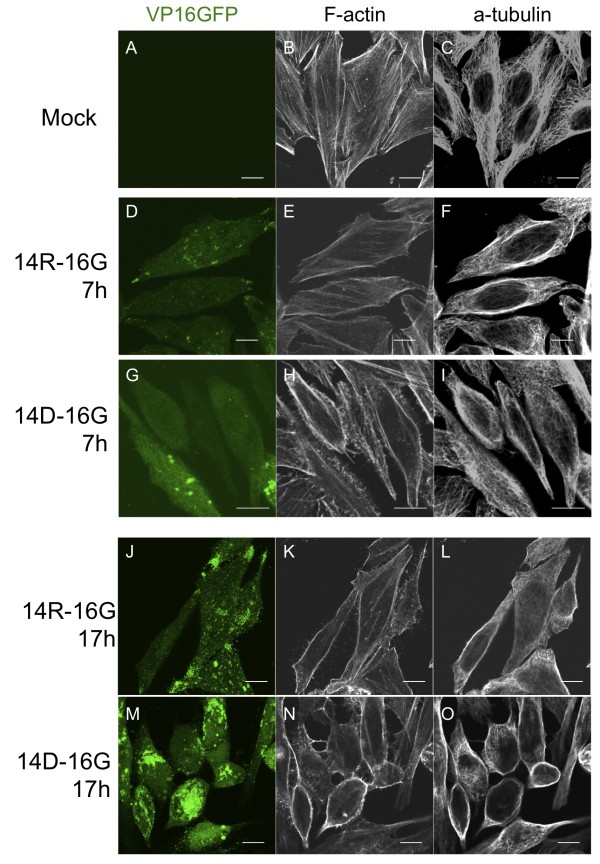
**Early alterations of cytoskeletal proteins in UL14D-infected cells**. HEp-2 cells were infected with wild-type and UL14D virus at an MOI of 3. Cells were fixed at the indicated time points, permeabilized and stained with Alexa Fluor^®^546-conjugated phalloidin and anti-α tubulin (diluted 1:1000). Following infection with UL14R and UL14D, actin stress fibers and the microtubule mesh became reduced from 7 h.p.i. in UL14D-VP16GFP-infected cells (H, I). At 17 h.p.i. actin stress fibers remained in UL14R (K), whereas UL14D-infected cells had no stress fibers and exhibited formation of cell-to-cell filamentous processes of actin (N).

There were several morphological changes seen in cells infected with UL14-defective virus at later time points after infection, including spherically shaped cells, shrinkage of the cytoplasmic volume, and an inward curvature of the nucleus (Figure 3E, F, K, L). At these time points, in cells infected with UL14D- or UL14(3P)-VP16GFP virus, the long axes of the cells gradually shortened from 11 h.p.i., but these changes were less apparent at 17 h.p.i. (Figure 3F, L). Therefore, we next investigated if virus infection could alter the distribution of cytoskeletal proteins using immunofluorescence analysis with phalloidin and an anti-α-tubulin antibody to detect filamentous actin (F-actin) and microtubules, respectively. In non-infected (mock) cells, there were many actin stress fibers with focal adhesions and a fine mesh of microtubules. Moreover, staining with anti-vinculin demonstrated focal adhesions present at both ends of the actin filaments (data not shown). In UL14R-VP16GFP-infected cells, many stress fibers were detected during infection, and small dots of actin were seen at 17 h.p.i. However, the number of stress fibers and focal adhesions were reduced as viral replication proceeded (Figure 6). Microtubules in UL14R-VP16GFP-infected cells maintained a mesh structure during infection, but unstrained filaments within the mesh were detected at 17 h.p.i. In cells infected with UL14D-VP16GFP, both the disruption of stress fibers and focal adhesions and the formation of cell-to-cell filamentous processes were detected in F-actin-stained cells. In addition, an unstrained structure of microtubules was detected at 7 h.p.i., and the microtubules were in a bundled or dotted structure at 17 h.p.i. These observations indicate that infection with virus encoding defective UL14 protein led to the disruption of cytoskeletal organization earlier than similar disruptions in wild type-infected cells.

### VP16GFP accumulated at aggresomes in UL14-defective virus-infected cells

**Figure 7:**
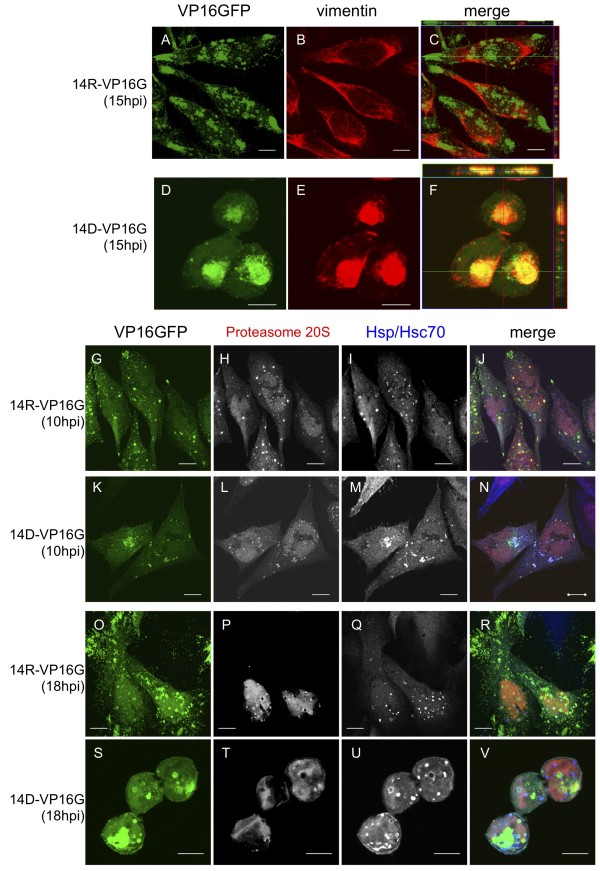
**Aggresome formation in UL14D-VP16GFP-infected virus, and protein quality control components in infected cells**. HEP-2 cells were infected with wild-type and UL14D virus at an MOI of 3. Cells were fixed at the indicated time points, permeabilized and stained with anti-vimentin, anti-Hsp70, and anti-protesome 20S subunit. VP16GFP localized to aggresomes surrounded by a vimentin cage in UL14D-infected cells (D-F), but the filamentous structure of vimentin was maintained in the UL14R-infected cells (B). At 10 h.p.i. when no aggresomes were detected in the UL14D-infected cells, the proteasome and Hsp70 localized to distinct punctate structures that partially colocalized with the VP16GFP foci in both Ul14R- and UL14D- infected cells (G-J, K-N). The VP16GFP foci localized to the periphery of cell nuclei (K-N). At 18 h.p.i. the VICE domains appeared in UL14R-infected cells, and substantial fractions of both the proteasome and Hsp70 localized to the nucleus (O-R). In cells infected with UL14D-VP16GFP, both the proteasome and Hsp70 were distributed in the cytoplasm and nuclei, and there was colocalization of Hsp70 and the proteasome with VP16GFP in the aggresomes (S-V).

We next investigated whether the sites of VP16GFP and UL46 staining were aggresomes where insoluble or misfolded proteins accumulate. A characteristic morphologic feature of aggresomes is the presence of a vimentin cage surrounding the accumulated proteins at the microtubule-organizing center [24]. Thus, we examined the vimentin structure in HSV-infected cells, and we observed a vimentin cage surrounding the accumulated VP16GFP in UL14D-VP16GFP-infected cells, but the vimentin in the UL14R-VP16GFP-infected cells maintained the cellular cytoskeletal structure (Figure 7). This observation suggests that the aggregated proteins, including VP16GFP and UL46, were isolated from the cell cytoplasm. Our results indirectly demonstrate that the VP16GFP and UL46 present in aggresomes were not associated with the capsid protein VP5 and, consequently, not used for viral replication (Figure 4). Before aggresome formation, misfolded proteins are either associated with molecular chaperones to prevent aggregation and/or facilitate refolding or degraded by the proteasome. However, molecular chaperones and proteasomes often accumulate around the aggresome [25]. Thus, we next examined the localization of the proteasome 20S subunit and the molecular chaperone Hsp70 in UL14R- or UL14D-VP16GFP-infected cells by immunofluorescence. In cells infected with UL14R-VP16GFP, the proteasome 20S subunit predominantly localized to the nucleus and a granular structure was seen in the cytoplasm at 10 h.p.i. (Figure 7). Hsp70 was distributed throughout the nucleus and cytoplasm, and small punctate structures were scattered around the cell. In cells infected with UL14D-VP16GFP, the quantitative distributions of both proteins were similar at 10 h.p.i., but the granular structure of the 20S subunit was not defined. Additionally, the Hsp70-positive structures colocalized with proteasome 20S and VP16GFP at the perinuclear region. At 18 h.p.i. the proteasome 20S subunit was almost completely localized within the nuclei of the cells infected with UL14R- or UL14D-VP16GFP, and Hsp70 formed ring structures within nuclei (Figure 7), which are defined as virus-induced chaperone-enriched (VICE) domains [26]. VICE domains were detected in both UL14R- and UL14D-VP16GFP-infected cells. In addition, some Hsp70 and the proteasome 20S subunit colocalized with VP16GFP in the aggresome, but only in the UL14D-VP16GFP-infected cells. The time course of the localization of Hsp70, proteasome, and VP16GFP in the cytoplasm of the UL14D-VP16GFP-infected cells suggests that misfolded and/or functionally impaired VP16GFP were associated with proteasomes and molecular chaperones. However, the increasing mass of accumulated VP16GFP by 18 h.p.i. exceeded the capacity of the cellular protein quality control (PQC) apparatus leading to the formation of aggresomes.

## Discussion

In present study, we showed that mutation of the HSP-like sequence of UL14 altered the intracellular compartmentalization of some HSV-1 tegument proteins, and induced the formation of aggresomes, which included VP16 and UL46, during the late phase of infection. The characteristics of UL14D-VP16GFP infection--including growth delay, early alteration of cell morphology, and aggresome formation--were clearly observed in cells infected with UL14(3P)-VP16GFP mutant virus, but these phenotypes were not observed in cells infected with UL14(K51M)-VP16GFP virus. These results suggest that a cluster of three charged residues (R60, R64, E68) within the HSP-like sequence of UL14, contribute to viral growth, cell morphology, and compartmentalization of tegument proteins. The _60_RLKSRARLE_68 _sequence of UL14, which contains a cluster of charged residues, demonstrates sequence similarity to a region of Hsp70 that forms an α-helix integrated in the substrate-binding region [8,9]. Mutation of the charged residues in UL14(3P) may disrupt the interaction of UL14 with other viral proteins, causing a loss of function. However, a direct interaction between UL14 and VP16 has not yet been demonstrated, and further analyses are needed to establish this model.

Immunofluorescence analysis identified several morphological differences between cells infected with UL14D- and UL14R-encoding viruses, and differences were primarily observed in the cytoplasm of cells. The nuclear distribution of the viral replication compartment, VP5 protein, VP16GFP, and VICE domains was unaffected by the deletion or mutation of UL14. Cunningham *et al*. previously reported that unenveloped capsids accumulated in the cytoplasm of UL14D-infected cells, and they suggested that UL14 facilitates the addition of the tegument and envelope to assembling virions [6]. Our results also indicate that UL14 is required for events following capsid formation.

The cytoplasmic aggregation of tegument proteins seen in UL14D-infected cells became pronounced as infection progressed. The synthesis of VP16GFP and UL46 proteins increased at approximately the same rate in cells infected with both wild-type and UL14D-encoding virus, as demonstrated by immunoblot analysis (data not shown). However, the growth kinetic analysis indicated that UL14D-VP16GFP-infected cells failed to produce infectious virions at the same rate as UL14R-VP16GFP. Moreover, in UL14D-VP16GFP-infected cells, tegument proteins were not efficiently incorporated into the virions. In a previous study, we showed that UL14 of HSV-2 mediated the translocation of both the capsid protein VP26 and the UL33 protein into the nucleus of cells in the absence of infection. UL14 protein also exhibited heat shock protein-like functions when singly expressed. Therefore, we hypothesized that UL14 in infected cells could also play a chaperone-like function and assist the translocation or functional maturation of some tegument proteins.

In this study, we found that HSV-infected cells eventually became rounded and exhibited a loss of adhesion to the extracellular matrix, but these changes became apparent earlier after infection with UL14D- or UL14(3P)-VP16GFP compared with rescued virus. In particular, we found that some actin stress fibers and focal adhesions of UL14D- and UL14(3P)-VP16GFP-infected cells were decreased at 11 h.p.i. and beyond, but these structures were maintained in mock- and rescued virus-infected cells. No actin polymerization-based protrusions or loss of focal adhesion complexes were seen in normal cells or those infected with rescued virus 11 h.p.i. Therefore, we speculate that UL14 has some role in maintaining the cytoskeletal organization in infected cells.

Aggregates of VP16GFP, UL46, and UL14(3P) were surround by vimentin and sequestered in the cytoplasm, indicating aggresome formation. Aggresomes are formed in cells when there is a large amount of misfolded or insoluble proteins accumulate with cells, but, under normal conditions, aggresome formation is prevented by the activity of the cellular protein quality control (PQC) machinery, which consists of molecular chaperones and proteasomes, which refold or degrade impaired peptides. In the nuclei of HSV-1-infected cells, Weller and colleagues showed that VICE domains contain cellular chaperones, proteasomal components and ubiquitinated proteins, and they suggested that VICE domains function as nuclear PQC centers to remodel or degrade aberrant nuclear proteins interfering with viral infection [27]. Large amounts of newly synthesized viral proteins are present in HSV-infected cells, and some likely require a functional PQC machinery, and these could be of viral and/or cellular origin. Thus, we hypothesize that the loss of UL14 function increases the amount of misfolded and/or insoluble proteins in the cytoplasm, because some late proteins in particular, such as VP16 and UL46, require the chaperone-like activity of UL14 to mature and/or properly localize within the cell.

## Conclusions

We speculate that the charged residues present in UL14 are important for its chaperone-like functions, and these residues are required for tegument protein maturation and contribute to the sophisticated HSV replication cycle. However, direct interactions between UL14 and VP16 or UL46 have not yet been demonstrated, and the molecular mechanisms underlying the observed cytoskeletal changes remain unclear.

## Methods

### Cells

The immortalized African green monkey kidney cell line Vero, the human larynx carcinoma cell line HEp-2, and rabbit skin cells (gifts from B. Roizman) were used. Cells were propagated in Dulbecco's modified Eagle's medium supplemented with 5% calf serum (Vero cells), 10% fetal bovine serum (HEp-2 cells), or 5% fetal bovine serum (rabbit skin cells), each supplemented with 100 U/ml penicillin, 100 mg/ml streptomycin, and 2 mM glutamine. All cells were maintained at 37°C with CO_2_.

### Viruses

HSV-1 wild-type strain 17 syn+, UL14-mutant virus UL14D, and rescued UL14R virus were kindly provided by C. Cunningham. HSV-1 strain MP44 (a syncytium-type 17syn+ VP16-green fluorescent protein [GFP] virus) was a gift from P. O'Hare. Viral stocks were prepared in Vero or rabbit skin cells, and were subsequently titrated separately on Vero cell monolayers. Growth curves were obtained as previously reported [6].

### Antibodies

The following commercial antibodies were used: mouse monoclonal anti-HSV1+HSV2 ICP5 (#ab6508; Abcam, Inc., Cambridge, MA), mouse monoclonal anti-HSV1 ICP8 (#ab20194; Abcam), Alexa Fluor^® ^546-conjugated phalloidin (#A22283; Invitrogen, Carlsbad, CA), mouse monoclonal anti-α-tubulin (#ab7291; Abcam), mouse monoclonal anti-vimentin clone V9 (Sigma, St. Louis, MO), rabbit polyclonal anti-proteasome 20S α5 (#ab11437; Abcam), mouse monoclonal anti-Hsp70 (#SPA-810; AssayDesign), Alexa Fluor^® ^555-conjugated goat anti-mouse IgG1, Alexa Fluor^® ^647-conjugated goat anti-mouse IgG1, Alexa Fluor^® ^555-conjugated goat anti-rabbit IgG, and Alexa Fluor^® ^647-conjugated goat anti-rabbit IgG.

### Immunofluorescence

Cells grown on coverslips were washed with phosphate-buffered saline (PBS) and fixed for 10 min in 4% paraformaldehyde in PBS at room temperature. For GFP visualization, the coverslips were mounted directly onto glass slides with PermaFluor (Thermo). For indirect immunofluorescence, fixed cells were permeabilized in 0.1% Triton X-100 in PBS for 5 min at room temperature. Each coverslip was inverted onto a droplet (20 ml) of blocking buffer (4% goat serum, 1% bovine serum albumin in PBS-Tween [0.05%]) on a clean Parafilm sheet for 30 min at room temperature. Primary and secondary antibodies (Alexa Fluor; Molecular Probes) were diluted in blocking buffer and reacted for 30 min at room temperature. To stain with two mouse primary antibodies at once, we used the Zenon^® ^Alexa Fluor^® ^labeling kit (Invitrogen). Samples were examined under a Zeiss LSM510 confocal immunofluorescence microscope.

### Viral replication kinetic assay

Analysis of one-step growth kinetics was performed as described [28] HEp-2 cells were infected with each virus at an MOI of 3 and incubated for 1 hour at 37°C to allow for virus adsorption. Any remaining extracellular virus was inactivated by a low-pH treatment (pH 3.0), and the cells were incubated at 37°C with 5% CO_2_. The culture medium was then replaced with newly prepared medium containing 2% fetal bovine serum. Cells and supernatants were harvested at the indicated times after infection. Virus progeny were titrated on Vero cells by plaque assays.

## Abbreviations

GFP: green fluorescence protein; h.p.i.: hours post-infection; HSP: heat shock protein; HSV: herpes simplex virus; IE: immediate-early; MOI: multiplicity of infection; PBS: phosphate buffered saline; PQC: protein quality control; UL14D: UL14-deleted; UL14(3P): three-point mutated (R60A, R64A, E68D) UL14; VICE: virus induced chaperone enriched.

## Competing interests

The authors declare that they have no competing interests.

## Authors' contributions

AO, YY, and YN designed the research, AO, YY, and YM performed the experimental work, AO conducted the data analysis and drafted the manuscript, and YY, YM, and HK participated in the data analysis and review of the manuscript. All authors read and approved the final manuscript.
